# Cognitive novelties, informational form, and structural-causal explanations

**DOI:** 10.1007/s11229-020-02585-4

**Published:** 2020-03-03

**Authors:** Andrew Buskell

**Affiliations:** Department of History and Philosophy of Science, University of Cambridge, Cambridge, UK

**Keywords:** Cognitive evolution, Cultural evolution, Novelties, Explanation

## Abstract

Recent work has established a framework for explaining the origin of cognitive novelties—qualitatively distinct cognitive traits—in human beings. This niche construction approach argues that humans engineer epistemic environments in ways that facilitate the ontogenetic and phylogenetic development of such novelties. I here argue that attention to the organized relations between content-carrying informational vehicles, or *informational form*, is key to a valuable explanatory strategy within this project, what I call *structural-causal* explanations. Drawing on recent work from Cecilia Heyes, and developing a case study around a novel mathematical capacity, I demonstrate how structural-causal explanations can contribute to the niche construction approach by underwriting the application of explanatory tools and generating new empirical targets.

## The niche construction approach

Human beings display a range of cognitive novelties—qualitatively distinct cognitive traits—as compared to our closest living ancestors. Among many such traits, the capacity to read, write, engage in mathematical reasoning, and attribute mental states, are substantially more sophisticated than what is found in other animals. In part because of their distinctiveness, the origin and development of cognitive novelties represent explanatory targets for researchers—and serve as ground zero for debates between nativist and empiricist accounts of cognition.

This paper develops one empiricist approach to explaining cognitive novelties. Absent any agreed-upon label, I call this the *niche construction approach*. Emphasizing the role of environmental activity, the niche construction approach holds that human beings engineer stable, information-rich, organized epistemic environments that facilitate the development of cognitive novelties. Though encompassing a range of scientific disciplines, heterogeneous methods, and various spatiotemporal scales, researchers adopting the approach nonetheless hold a number of common assumptions. These are:

1. That powerful evolutionary feedback loops select for traits that can exploit and engineer informational domains. Starting with a cognitive profile much like extant *Panins*, only small tweaks to social tolerance, executive control, working memory, and attention are needed to facilitate an increased reliance on social learning.^1^ Such social learning could in turn co-evolve with the increasing availability of information generated by conspecifics.^2^ This dynamic feedback loop could further exaggerate the sociality and cognitive flexibility of ancient hominins, amplifying the sophistication, organization, and effects of activity through modified life history traits (e.g. longer adolescences, increased reliance on alloparenting), means of resource capture and processing (e.g. distributed systems of labor, sophisticated collaborative hunting), and economic exchange (e.g. extended kinship).^3^
2. An explanatory emphasis on epistemic features of human cognition and the world: human beings are characterized as epistemically opportunistic agents who flexibly adopt strategies and exploit cues to extract high-quality information relative to their goals. Information is interpreted broadly as the epistemically salient aspects of learning and teaching, object properties, and scenarios—and is thus carried by varied material vehicles (speech, bodily movements, artefacts, etc.).^4^ This inclusive characterization allows for investigation into ways agents and heterogeneous information-carrying vehicles together constitute salient informational assemblages. Such assemblages may be ephemeral (as when teachers simplify actions or problems to suit the abilities of learners (Flynn et al. 2013)), persist for days or weeks (as when individuals leave material cast-offs or trace evidence in the environment (Sterelny 2012)), or generate enduring organized structures (as with language (Clark 1997) or artefacts (Hutchins 1995)).
3. Lastly, an evolutionary narrative whereby increasingly sophisticated human cognition is tied to capacities for engineering and exploiting structured informational domains. Here, relationships between social and technological change lead to increasingly enriched and organized informational environments. So, for instance, increased population size can create variation in the quality of informational sources, providing opportunities for learners to attend to more capable models, and thus facilitating the dissemination and improvement of skills and techniques. Enlarged population sizes can also support expanded suites of tools and techniques that can be combined and recombined to increase and improve technologies.^5^ Improved skills and technologies in turn can facilitate greater resource capture and, as a result, larger population sizes that support even better skills, tools and technologies. As effective population sizes increase and the sophistication of skills and technologies improve, hominins gain increasing ability to change both their lifeways and the world; transforming their social organization, strategies of trophic exchange, bodies of knowledge, and technological sophistication.

I think the niche construction picture of human evolution, development, and cognition is powerful and compelling. By emphasizing the ways in which human activities can enrich their epistemic environments—and in so doing, set off a number of powerful feedback relationships—the approach represents an important and plausible alternative to nativist accounts of human cognitive evolution (e.g. Barrett 2015).

Circumventing this entrenched debate, my present aim is to develop a powerful, as yet insufficiently articulated, explanatory strategy available within the niche construction approach. This *structural-causal* strategy explains the development of cognitive novelties by showing how an individual’s inferences and manipulations can come to reflect the organized relationships holding between informational vehicles in their environment. In other words, cognition can come to mirror the structure, or *informational form*, of organized assemblages.

The structural-causal strategy draws on work from embodied and distributed cognition, cognitive ecology, and ecological psychology.^6^ One key insight of these literatures is that the organization and flow of information is important for understanding both the development of cognition and its occurrent functioning. Structured informational domains like material artefacts, linguistic systems, or workshop apprenticeships can facilitate the acquisition of abilities and regulate their deployment—and here I extend this point to help explain cognitive novelties. Nonetheless, this paper is not intended as a contribution to these literatures. My focus is on cognitive capacities whose mature functionings are not constitutively reliant upon external scaffolding.^7^ This leaves open the possibility that such capacities may involve a substantial embodied component—a possibility I return to later. Still, there is a contrast to be drawn between the capacities at issue here and the many examples in the above literatures where capacities are constitutively reliant upon ongoing engagement with a structured world. My focus is on the former because they provide striking examples of persistent cognitive reorganization, and because the core targets of the niche construction approach—at least mind reading, language, selective social learning, and imitation—are taken to be instances of such persistent cognitive reorganization.

My strategy for articulating informational form and its role in structural-causal explanations of cognitive novelties begins by engaging with Cecilia Heyes. Heyes’s recent work has developed a powerful model for explaining cognitive novelties using the mechanisms of cultural evolution. Though Heyes is something of an uncomfortable bedfellow with the niche construction approach, her work bears several of its hallmarks, notably: the appeal to structured information domains embedded in material and social props, the iterative improvement of such domains through deliberate activity, and the role of discriminating epistemic agents.^8^ Because of this, exploring Heyes’s machinery is valuable for expanding the reach of the niche construction approach—indeed, one way of understanding this paper is as an attempt to analyze and integrate the two.

I begin by outlining Heyes and her work on the cultural evolution of cognitive novelties. As I suggest, her framework presents a puzzle; by strictly distinguishing cognitive hardware from informational input, Heyes undercuts a substantive role for exogenous information in the development of new cognitive functioning. Yet by exploring a case study in mathematical cognition and Heyes’ own work on imitation, a different picture emerges: one where learning can lead a mirroring of the structured organization of information in the world. In the final sections, I suggest what model of cognition might underpin this learning, and explore how structural-causal explanations might be developed further.

## Culturally evolved cognitive novelties

The work of Heyes (2012a, b, 2018, 2019) is at the forefront of explaining the evolution of distinctively human cognitive novelties. Before I begin sketching her approach, I note that Heyes’s work is expansive. It marshals a range of empirical research, develops new conceptual machinery, and touches upon issues in many literatures. I thus cannot give a comprehensive treatment of her account and focus on those aspects central to the explanation of cognitive novelties.

Heyes’s targets are novelties deep within the hominin lineage—what she calls ‘cognitive gadgets’—that support cumulative and adaptive cultural evolution.^9^ Like niche construction researchers, Heyes argues that only small changes to ape-like cognition were needed to kickstart evolutionary processes culminating in cognitive novelties. Like niche construction researchers, Heyes believes that such evolutionary processes were amplified as hominins increasingly came to rely on social learning and collective enterprise within groups. And like niche construction researchers, Heyes argues that cultural evolutionary processes can create sophisticated and adaptive bodies of skills, tools, technologies, and institutions. What is distinctive about Heyes is her claim that cultural evolution can generate changes in cognitive functioning—that cultural evolution can generate cognitive novelties.

At this point, it is helpful to step back and distinguish two mechanisms by which cultural evolution might possibly change cognitive machinery. The first is gene-culture coevolution. This occurs when cultural practices modify selection pressures, leading to shifts in genetic frequencies. The second occurs when cultural evolution processes (selective social learning, imitation, cultural recombination) lead to cumulative change in the adaptiveness of practices and technologies. Niche construction researchers appeal to both mechanisms when explaining human cognitive evolution. Heyes’ model, however, shows the power of the latter: mechanisms of cultural evolution themselves might explain the origin and improvement of cognitive novelties—no change in genes required.

Heyes sees cultural evolution as strongly analogous to biological evolution. This approach—what she calls a ‘Campbellian’ after the work of Campbell (1965)—assumes that cultural variation is constantly produced, is random with respect to fitness, and that fitness determines downstream frequencies of cultural traits in populations (Heyes 2018, pp. 32–36). Here, ‘fitness’ is indexed to cultural traits and measures their effects on the relative reproductive (that is, biological) success of individuals. Lastly, Heyes takes mechanisms of cultural group selection and cultural diffusion to be the predominant means by which cultural traits spread (*ibid*., pp. 198–203).^10^ Taken together, these elements provide a schematic representation of what Heyes calls a ‘force theory’; an idealized sketch of the salient causal processes of cultural evolution that can generate cognitive novelties responsible for downstream cumulative cultural evolution.

There are two important epistemic and conceptual moves underpinning this schematic that are worth drawing out. The first is an explanatory focus on the adaptive role of such mechanisms. As Heyes notes, she is “interested in adaptedness, why some cognitive mechanisms do their jobs better than others.” (2019, p. 2) What is important is not just the evolved function of such capacities, but also how different variants of such mechanisms do better or worse at fulfilling such a function, and thus increase or decrease biological fitness. As mentioned above, Heyes appeals to Campbellian cultural evolution and cultural group selection as mechanisms for explaining such adaptiveness. I pick up and analyze this adaptationist line of thought in the next section.

The second important move concerns individuation and the conceptualization of cognition; the means of differentiating cognitive novelties for empirical research. Heyes sees contemporary accounts of cognitive science as providing such means. As she writes:

cognitive mechanisms (mills) are unitized by cognitive science, and, within the framework of cognitive science, different versions of a mechanism—different variants—can be distinguished according to what they do and how they do it; the kind of information they process, and the computations they use (Heyes 2018, p. 38).

Roughly, cognitive science provides the means for identifying different mechanisms, their function, and the kind of information that these mechanisms typically process.

In adopting the strategy, Heyes takes on board a standard distinction between cognitive mechanisms and their informational inputs: cognitive novelties are cognitive hardware that are functionally individuated by cognitive scientists and that perform operations on information-bearing vehicles of content (the inputs).

The contrast between mechanistic functioning and informational inputs is embedded in Heyes’s distinction between ‘grist’ and ‘mills’. Grist is information expressed or embodied in behavior: the use of a particular tool, style of dress, notion of appropriateness, or the like. It is “what we do and make, and the contents of thought” (2019, p. 2), what can be “‘taken in’ (and produced by) human minds.” (2018, p. 37) Mills, by contrast, are cognitive mechanisms. As noted above, these are individuated by cognitive science according to the specific function they underwrite, and variants of these can be better or worse at their job.^11^

The mechanism/input distinction generates a puzzle. Heyes argues that cultural evolution is a process that selects for fit, information-bearing vehicles that generate novel cognitive mechanisms. By adopting a mechanism/input distinction, however, Heyes undercuts the plausibility of that picture; for if information can only be an input to a mechanism, then how could it influence the development of that mechanism? Heyes is committed to the claim that we learn mindreading, imitation, and rules for social learning—but the conceptual machinery of her account makes mysterious this banner claim.

Let me put this another way. What Heyes requires is an account of cognition and cultural evolution whereby cognitive novelties emerge from learning processes sensitive to the organized structure of informational domains. But equating novelties with the brain-bound mechanisms of mature individuals directs attention away from such processes, and suggests that exogenous information only factors as grist to canalized mechanistic mills.

Can the organization of exogenous information play such a difference making role in generating cognitive novelties? I think that it can. To make space for such a role, I first turn to consider how cognitive novelties should be conceived.

## Cognitive novelties and informational form

Cognitive novelties are one kind of evolutionary novelty; qualitatively distinct complexes of form and function (Love 2008).^12^ To adopt a maximally inclusive formulation, ‘functions’ are relationships of means-ends fittedness between a system and purposes, while ‘form’ consists in the structural relationships, or organization, among elements composing such a system. Under this permissive formulation, evolutionary novelties can emerge, and indeed are found, at varying scales from the proteomic to the morphological.

The identification of novelties is sensitive to description. Novelties described from one angle may not be so from another. This is a feature and not a bug. Such description-dependence underwrites the comparative logic by which evolutionary researchers distinguish, situate, and explain novelties using relevant contrast groups. For most comparisons, biologists appeal to close phylogenetic neighbors at the same taxonomic rank. Thus, elephant trunks are novelties among ungulates, and feathers among chordates.

Novelties are interesting empirical targets because they mark off different ways of living; supporting distinct capacities and reflecting unique evolutionary histories. And while researching putatively unique traits requires scientific ingenuity, researchers ask fundamentally the same set of questions about novelties as they do of any other trait: ‘how did it evolve?’, ‘how does it typically develop?’, and ‘what is its function?’. The same questions apply to human cognitive novelties: novelties are compelling empirical targets because they support distinctive human lifeways.^13^ They are also tricky to study because of their putative uniqueness. Nonetheless researchers can and do investigate them, employing a wide range of theoretical and empirical strategies to understand their origin and evolution.

We saw above Heyes’s strategy for approaching these empirical questions: cognitive science individuates novelties by identifying their functions, while cultural evolution (particularly group selection) is the mechanism by which means-ends fittedness can be improved over time.

A functionalist and adaptationist approach is not the only way to pursue evolutionary and developmental questions about novelties, however. Evolutionary developmental biologists, for instance, emphasize organismic form and the role of constraints in facilitating viable and adaptive organisms. They do so by adopting a range of formal and empirical methods, identifying how heterogeneous elements (genes, transcription factors) enter into dynamic causal relationships where activation profiles play a crucial explanatory role.^14^ The main takeaway for current purposes is that valuable understandings of traits can result when they are understood as emerging from an underlying system of organized elements—many of which only exert an influence for a short period of time.

Here I argue that an analogous approach can be used to explain the cultural evolution of cognitive novelties. The general idea is that developmental environments contain heterogeneous vehicles of informational content (external artefacts, linguistic labels, the behavior of other agents) organized in systems. And just like in cases of evolutionary developmental biology, the structural relationships between these vehicles can make a difference to the development of cognitive skills, even if they are only present for short periods of time.

To help make these points more intuitive, I develop a case study that draws attention to the difference-making role of this informational form in structural-causal explanations. In this example, informational content is important but insufficient to explain the development and operation of the cognitive capacity at stake. Attention to the organization of information-bearing vehicles in the material nature of external props, bodily movements, and long periods of effortful learning is also required. After exploring this case study, I return to consider structural-causal explanations and informational form in more general terms.

### Abacus-based mental calculation

The case study looks at abacus-based mental calculation (AMC), an impressive capacity where skilled practitioners manipulate mental representations of abaci to efficiently solve mathematical equations. One of the remarkable features of such skilled practitioners is the speed and reliability of their calculations. Priyanshi Somani, for instance, is an exceptionally skilled AMC user who won the overall title at the Mental Calculation World Cup in 2010. This achievement is impressive given the difficulty of the tasks: the addition trial alone required computing ten questions with ten ten-digit summands in under three minutes.^15^ AMC is extraordinary—but how does it come about?

Using abaci as part of mathematical instruction is standard practice across a number of Central and East Asian communities. But developing capacities for AMC requires instruction, practice, and commitment beyond standard education. Frank and Barner (2012) for instance, carried out ethnographic and experimental studies on AMC students in Gujarat province, India, where the program is a three-year after school course.

**Fig. 1:**
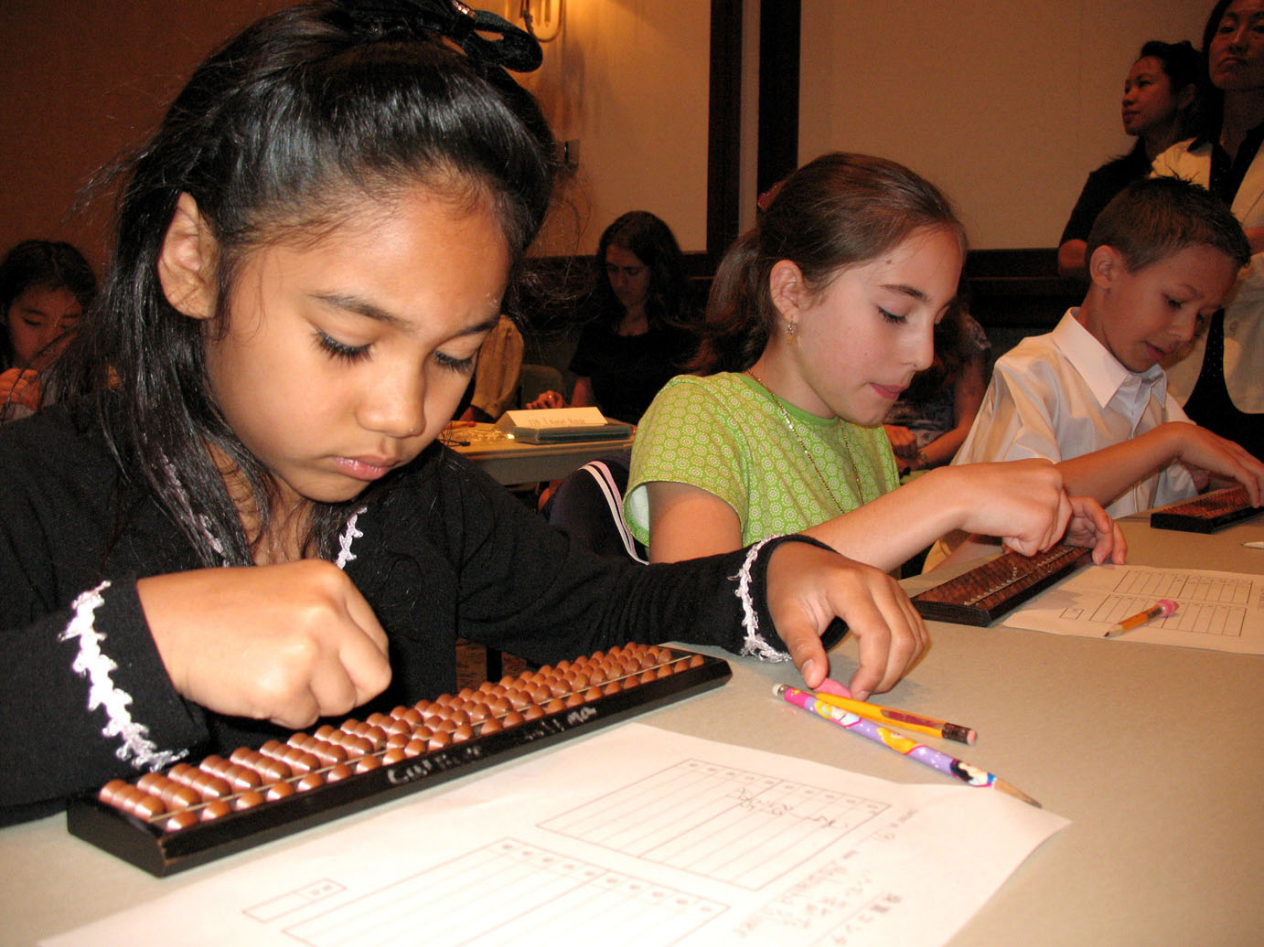
Children completing at the 25th Annual Soroban Contest in Tokyo. Public Domain Image (United States Air Force)

The most widely used abacus in these programs is the Japanese soroban abacus (Fig. 1). This abacus is divided into two horizontal sections, separated by a beam. Run through this beam are a number of bead columns, which typically represent increasing base ten units (ones, tens, hundreds, etc.). The bottom section—below the horizontal beam—contains four ‘earthly’ beads, each representing one decimal (base ten) unit of the respective column. The upper section contains a single ‘heavenly’ bead, representing a quinary (base five) unit. When beads are moved towards the horizontal dividing beam they are put ‘into play’, and represent a particular numerical quantity.

The material character of the abacus is important for a number of reasons. The first has to do with the general representational character of abaci. Representing numerical quantities with beads is manifestly different than numerical notation (e.g. the inscription ‘22’) even if the quantities represented are the same. These representational differences are particularly salient with regards to calculating algorithms. Carrying out mathematical operations (e.g. addition, multiplication) on abaci means learning a different set of algorithms than those involved in carrying out the same operations using pen and paper (Overmann 2018). A second important material feature of abaci is that they are reliable tools. Unless someone jostles or shakes the abacus during the process of calculation, the abacus can be reliably manipulated through intermediate steps to complete algorithms. Third, algorithms involve the bodily manipulation of material beads, and these manipulations can be made fluid and transparent over time. Though learning such algorithms may have initially involved deliberate movements and the double-checking of results, successive practice can routinize these actions until they are effortlessly exercised.

Although the use of abaci can involve routinized movements, this does not mean that using the abacus is trivial, or that all cognitive operations can be automatized. Consider another problem Somani had to solve at the Mental Calculation World Cup: computing a cubed root. The algorithm for carrying out such a calculation on an abacus is demanding. Even with a physical abacus, the algorithm requires the user to mentally subdivide bead columns into three distinct sections, partition the target number into three-digit tuples, and perform iterative cycles of division and multiplication. Nonetheless, despite the complexity of the algorithm, effortful practice can decrease cognitive demands by routinizing the bodily manipulations required within each section and partition.

What is extraordinary about AMC, however, is skilled practitioners can carry out all the same calculations and algorithms as normal abacus users *in the absence of an external abacus*. AMC practitioners internalize the content carrying vehicles, manipulating visual representations of beads and their movements in working memory.

Three pieces of evidence support the claim that AMC practitioners manipulate imagistic representations abaci in working memory. The first is that AMC practitioners have access to the intermediate stages of computation. Stigler and colleagues (1986; Stigler 1984), for instance, found that AMC practitioners were able to identify whether a particular arrangement of beads would be moved through when solving a particular problem. The second is that AMC experts are much more likely than non-abacus-using calculators to make computational errors involving multiples of five. This, they suggest, is because AMC users internalize a representation of the ‘heavenly’ bead—which, recall, represents a quinary (‘base five’) unit—that when erroneously manipulated can explain the effect.

Lastly, Frank and Barner (2012) introduced distractor tasks to interrogate the nature of the representational content—with AMC practitioners and controls either carrying out verbal (repeating a story) or motoric (drumming fingers) distractor tasks, while requiring subjects to mentally compute two-digit summands. All AMC practitioners performed better than controls carrying out mental addition, and some were able to perform perfectly. This can be explained if AMC calculators are manipulating imagistic representations of abaci rather than linguistic ones.

But what exactly do these manipulations consist in? One hint comes from looking at the role of motor behavior in calculations made with the abacus present. Donlan and Wu (2017) show that the number of operations using complementary numbers in different bases—that is, those involving manipulations of both the quinary (base five) ‘heavenly’ bead and decimal (base ten) ‘earthly’ beads—correlates with solving speed. Both expert and non-expert abacus users are slower to solve problems when they involve more complementary number operations. This demonstrates that even when motor patterns underlying algorithms are mastered, they are not automatized, and continue to require monitoring and control. (Fridland 2014, 2017).

When one turns to consider AMC, bodily manipulations continue to play an important role: AMC calculators continue to make finger movements and gestures even when manipulating mental abaci; these gestures are exaggerated as tasks become more difficult, and; motor interference tasks do have a negative influence on performance (Frank and Barner 2012; Brooks et al. 2014). This suggests that motor behavior plays non-trivial role in the actualization of AMC abilities. This is unsurprising: abaci algorithms are constituted by the movement patterns of beads. Even when manipulating imagined abaci, the manipulation of beads seem to be in accordance with the manipulations that would have taken place on physical ones.

To sum up, AMC is a cognitive novelty: it represents a distinct kind of mathematical capacity. It can be applied to a range of mathematical tasks in an incredibly impressive and robust manner. Yet the development and operation of these capacities is seemingly best explained by learners acquiring standards and motor routines for manipulating organized, information-bearing material vehicles. AMC practitioners further learn to apply these standards and motor patterns to imagistic representations of these vehicles through further effortful training.

To put it bluntly, the development of AMC capacities cannot be explained by pointing to informational content alone—the quantitative content that the beads represent. As the above account makes clear, material, bodily, cognitive elements and their interrelation are needed. Over hundreds of hours of experience, first with real abaci, and then increasingly without, individuals cultivate embodied skills for manipulating abaci. These same skills are then used to manipulate imagistic representations that mirror the material organization of abaci beads, and may be increasingly routinized, though not automatized, over time. Explanations for AMC need to appeal to the material character of the soroban abacus (particularly its combination of quinary- and decimal-base beads grouped in columns), the development of specific bodily skills, the nature of representations manipulated in working memory, and long effortful learning and internalization.

### Further reflections on form

The case study on AMC is an evocative illustration of what I mean by ‘informational form’: here, the structured information embedded in the organization of the soroban abacus. It also illustrates how structural-causal explanations work. Over hundreds of hours of practice, the cognition of AMC practitioners comes to mirror the structural features of the abacus. But how might these explanations work more generally? How can the organization of informational domains play a difference making role in producing cognitive novelties?

Recall above I identified a feature of Heyes’s account that renders the relationship between exogenous information and the development of cognitive novelties mysterious: a strict separation between cognitive mechanisms and informational input. This seems to undermine a positive role for exogenous information in the development of cognitive mechanisms. I also noted that Heyes focuses on adaptation, highlighting how different mechanisms can be better or worse at fulfilling specific functions.

The above case study reveals that for at least some cognitive novelties, a synchronic functionalist perspective can obscure important causal factors during development. This is because (as is familiar from work in evolutionary developmental biology) elements critical for trait development may be ephemeral and not present in mature traits. Thus, while a physical soroban abacus is crucial for developing AMC, practitioners do not need one once they have achieved proficiency. A focus on the synchronic functioning of hardwired mechanisms would thus miss the crucial developmental role of external props (Clark 2008).

Just as importantly, the above case study show that learners grasp of the organized relationships between the material vehicles of information is key to explaining the capacities they develop. Agents acquire more than bare informational content—more than just a mapping relationship between bead positions and numerical quantities. AMC practitioners acquire the ability to recognize and manipulate internalized vehicles of information, and to do so in ways that embody some standard of correctness. The routinized skills they acquired to manipulate physical abaci are put to use manipulating imagined ones.

As such, the structural-causal strategy explains cognitive novelties diachronically: emphasizing both extended periods of development and the possibility of important yet transitory elements. It also emphasizes informational form in the sense that the cognitive mechanisms develop a similar ‘shape’ or structure to that of the informational assemblage agents engage with. Form in this sense is meant to echo how the term is used in evolutionary developmental biology.

Importantly, such an explanatory approach does not repudiate functionalist or adaptationist approaches to the evolution and development of novelties. The two may in fact be complementary: a focus on the mirroring of form illuminates how structured elements and relationships serve as plausible targets for selective processes. Such complementarity is familiar from the philosophy of biology. Calcott’s (2009) *lineage* explanations, for instance, demonstrate how organized systems (his ‘biological mechanisms’) are productive constraints on natural selection, limiting viable variation. The elements and organization of these systems are nonetheless the targets for adaptive change. As Calcott argues, it is the combination of two explanatory strategies—developmental and evolutionary—that best explains how gradual changes to an underlying system lead to the eventual production of specific novel phenotypes.

The moral is that including structural-causal explanations in one’s tool kit does not mean taking out all the adaptationist ones. The two are not exclusive. Understood on the model of lineage explanations, the structural-causal explanations of cognitive novelties articulate the (sometimes ephemeral) elements and relationships that constrain the development and evolution of cognitive novelties. Taken together with our best theories of cultural and biological evolution, such a strategy can help to explain the possible and actual evolutionary trajectories of human cognition.

## Exogenous information and cognitive novelties

With these resources in hand, I return to consider the motivating puzzle of the second section—what is the relationship between exogenous information and cognition such that the former can cause transformative change in the latter? Here I consider and reject two prominent formulations from the cultural evolutionary and distributed cognition literatures. In the next section, I’ll return to Heyes and suggest that her work on imitation provides a promising way forward, despite the methodological and conceptual caveats made above.

The two formulations I consider in this section share a common feature: that the environment serves as a *repository* of information, and that what is interesting about exogenous information is the *quantity* of information that can be stored. Unsurprisingly, an overarching concern with the quantity of information tends to downplays the importance of how such information is organized.

The first formulation takes exogenous information to be predominantly symbolic, and is found in the environment where individuals have made inscriptions on objects. The content of such inscriptions can later be recovered by means of interpretation. This is a view familiar in mainstream work within the cultural evolutionary literature. Boyd and Richerson (2005), for instance defend the view that while “some cultural information is stored in artifacts,” they are quick to assert that without “writing […] artifacts can’t store much information” (61) This view is echoed by Mesoudi (2011, p. 3) who makes the point more explicit: externalized information is embedded in “extrasomatic codes such as written language, binary computer code, and musical notation.” Given that symbolic extrasomatic codes are a recent human invention, this means that for much of human evolution “the most important aspects of culture still tend to be those stored in our heads.” (Richerson and Boyd 2005, p. 62).

The second prevalent formulation of exogenous information takes it to consist in cues or evidence—either about the state of the world, or a relevant set of causes—that are recovered through processes of inference. This approach sees information in the world as available for use in the orientation, guidance, and fluid deployment of behavior. This guidance might be mundane; as ordinary as following in previously set-down footpaths through heavy brush. Yet it can also be more complex and involved, as when one uses a spear-thrower as a template for making another spear-thrower. (Sterelny 2010) Distributed cognition theorists have long been impressed by this on-demand interaction with information in the world. Their examples—for example, of distributed memory strategies (Tribble 2005; Sutton 2010) and the savvy use of physical manipulation (Clark 1997)—reveal how human beings *offload* information onto people, places, and things, in ways that can influence later behavior.

The two approaches depict the relationship between exogenous information and cognition differently. For Boyd and Richerson, and indeed for many other cultural evolutionary researchers, information is externalized through deliberate inscription and storage. Information repositories can later be consulted, double-checked, and amended. Yet the implication of this view is that one cannot store a great deal of it until the development of a combinatory and recursively structured code, as with written forms of language.

By contrast, the views of Sterelny and distributed cognition theorists hold that exogenous information is widespread—visible in the many traces of successful and unsuccessful human action in the world. This sees exogenous information as available for guiding future behavior, prompting the deployment of skills, and even being internalized into cognitive routines. Putting it evocatively, this is view that Andy Clark describes as being “*promiscuously* body-and-world exploiting [[…]] forever testing and exploring the possibilities for incorporating new resources and structures deep into their embodied acting and problem-solving regimes” (Clark 2008, p. 42).

Empirical evidence supports the importance of both formulations, yet recent work has tended to emphasize the importance of the latter, distributed view of information (Clark 2008; Sterelny 2012; Malafouris 2013). While human beings did and do inscribe information, it is not the case that environments were information-poor until the widespread use of symbolic inscriptions. Indeed, the fluid and flexible use of exogenous information extends deep into hominin history. Human beings of a hundred thousand years ago had already developed sophisticated technological toolkits, control of fire, collaborative hunting practices, tracking techniques, and stoneknapping skills. And modern accounts describing the development of these skills emphasize how they require long, effortful practice, picking up on cues and information from teachers, the environment, and the artefacts themselves (e.g. Wadley 2010; Hiscock 2014).

Nonetheless, even on this richer understanding, it is hard to recover a route by which exogenous information plays a difference-making role in the development of cognitive novelties. If anything, it makes the puzzle even harder. For if human beings are constantly engaged in offloading information onto the environment, perhaps interacting with these resources in clever on-demand ways, it is hard to see how this would later translate into radically altered cognitive machinery. Though distributed cognition researchers are right to hype the various ways in which human beings cleverly interact and exploit the world—often increasing performance capabilities and decreasing performance demands—this is not yet an account of how novel cognitive traits come about.

## Imitation, associative sequence learning, and form

In several areas of Heyes’s work, she follows something like the distributed view of informational offloading: culturally evolved technologies, teaching strategies, social interaction, and environmental cues guide infants in the acquisition of novel cognitive functioning. Exogenous information, on this picture, provides reliable and readily available scaffolding that facilitates the acquisition of cognitive novelties.^16^ Yet when Heyes turns to consider imitation—the cognitive novelty which she herself has spent a great deal of time investigating (i.e. Heyes 2001; Catmur et al. 2009; Ray and Heyes 2011)—a distinct kind of relationship between exogenous information and cognition emerges.

Imitation is the capacity for the faithful copying of another’s observed bodily movements. This is a cognitively sophisticated capacity requiring topographic mapping from the observed actions of agents onto first-person sensorimotor experiences. This is non-trivial, and the current indication is that human beings are unique in the sophistication of and their reliance upon imitation.^17^

Heyes’s account for the emergence of topographic mapping capacities is her Associative Sequence Learning (‘ASL’) model. On this model, topographic mappings are produced when observed behavior becomes linked to first-person sensorimotor experiences underwriting similar behavior. These links are ‘vertical associations’, a “sensory representation of an action linked to a motor representation of the same action”. (Heyes 2018, p. 107) Such links can be made and strengthened according to standard models of associative learning. As learners develop, they require more and more fine-grained vertical associations, gradually acquiring more and more competence in mimicking behavior.^18^

In explaining how and why such vertical associations are prevalent enough such that human infants reliably acquire imitation, Heyes employs the tools of the niche construction approach. One route for establishing vertical associations runs from teachers and parents to children. Caregivers often mime the sounds, movements, and expressions of infants—providing the contingent temporal association between infant’s sensorimotor experience and observed behavior. Another route runs between infants and cultural props. Mirrors, and perhaps other artefacts, can similarly provide sensorimotor feedback for forming vertical associations.

One implication of Heyes’s account is that, were vertical associations to change, different kinds of topographic mapping functions would occur. That is, if a learner were to systematically associate raising their left hand when a model raises their right hand, they would form a topographic relationship between the two. Here, the input/output profile of associative actions is systematically changed, leading to a modified suite of vertical associations. Experimental results support this supposition. Even spontaneous ‘automatic’ imitation, where observed behavior leads to the unreflective production of the same behavior, can be trained away or caused to generate ‘counter-imitation’—not unlike raising my left arm in response to you raising your right arm (Heyes et al. 2005; Catmur et al. 2009). As Heyes (2018) notes, “imitation can be diverted by relatively brief periods of sensorimotor learning, in which people are exposed to mismatched and arbitrary relations between observed and executed actions” (116).

This is important. Whether or not the ASL model is a complete account of imitation—or merely a sketch of one central component (Subiaul 2016)—it embodies a radically different kind of relationship between exogenous information and cognitive mechanisms. According to the ASL model, there is a direct correspondence between input/output profile of observed action/sensorimotor behavior and the functional capacity of agents. Indeed, Heyes account suggests that the two are identical: distinct training regimes generate distinct topographic mappings.

This picture of information repudiates Heyes’s distinction between grist and mills. Recall that for Heyes, grist are discrete ideas, practices, and technologies: “what we do and make, and the contents of thought” (2019, p. 2). Grist are distinct from mills, or cognitive mechanisms, which describe “the way or minds work” (*ibid*.) As I said above, this is a picture amenable to the idea of information offloading—that the world is rich in information which can be reconstructed and inferred by the grinding mills of mentation. But it is also a picture where the relationship among vehicles of information is not seen as relevant to developmental explanations of cognitive capacities. Information is just out there in the world, waiting to be fed into cognitive mechanisms. All this changes on the ASL model. Here the grist of exogenous information—one’s reflection in mirrors, the mimicking behavior of parents and caregivers—end up creating a profile of observed actions linked to sensorimotor experience. It is attention to this informational form, the relationships holding between observed action and sensorimotor experience, that is needed to explain the emergence of imitative capacities.

## Skills and internalization

Heyes study of imitation is useful for understanding how exogenous information can factor into explanations of cognitive novelties. Of course, the ASL model is a simple one, operating at very low level of perceptual and sensorimotor processing. As such it will not always be a perfect fit for understanding how informational form can factor into explanations of other cognitive novelties. AMC is case in point; explanations of it seemingly require pointing to internalized imagistic representations and fluidly executed motor routines. Still, I think the model is helpful. Here I want to outline how the examples marshalled up to this point help to illuminate the cognitive capacities underwriting structural-causal explanations.

First, the ASL model further reinforces the need to consider cognitive novelties diachronically, as generated through temporally extended developmental regimes. Moreover, as the niche construction approach makes clear, such regimes can involve interaction with a wide range of cultural props, deliberate teaching, and structured environments, all of which can facilitate the transfer of information. And as stated above, a diachronic perspective may also be needed to identify the necessary but ephemeral role of heterogeneous elements.

Secondly, the ASL model directs attention onto agents’ grasp of the relationships holding between elements in a structured informational domain. Cognitive novelties can emerge when agents’ cognitive systems come to mirror the exogenous informational domain, and when such agents acquire the standards for manipulating and engaging with such domains. With AMC, these are inculcated by giving simple examples, problem sets, and constructive feedback. Practitioners are taken to be competent when they can reliably produce correct answers to mathematical queries, and explain the steps and reasons for having such answers.

Third, these acquisition processes are demanding, and this explains why long periods of effortful practice are often required for developing cognitive novelties. Though it seems mundane, this is an important point, and in focusing attention on effortful practice I am explicitly linking the development of cognitive novelties to the literature within the niche construction account that emphasizes the role of skill in human cognitive evolution (Sterelny 2012).

Birch’s (*mans.*) skill hypothesis, for instance, holds that hominin cognitive evolution was driven in part by capacities for complex motor and craft skills. As Birch argues, there is good archaeological evidence for the deep history of skill in the hominin lineage; especially for the construction of sophisticated lithic tools like Acheulian bifaces. Constructing such tools requires the iterative application of knapping techniques, evaluation of on-going progress, and insight into the form and function of the final product. On Birch’s account, the deployment of skills is underpinned by *cognitive control models*. These dynamic models represent the causal features of the skill domain, anticipate the effects of sensorimotor activity, and respond to mismatches when they occur. To use Birch’s language, responses to mismatches encode *standards of correct performance*: representations of the expected dynamics of a causal domain and means to bring those dynamics back into line.

Given the nature of the paleoanthropological evidence available, Birch develops his account using examples of motor skill. Yet he speculates that the skill hypothesis may have a broad explanatory remit; arguing that the selection for skill may explain the origin of human norm psychology. As he suggests, only small tweaks would have been needed to expand cognitive control models to explain how standards of correct performance might be applied to social phenomena like collaborative hunting, kinship norms, and resource management. If Birch is right, then the ubiquity of human norms may in part be explained by an expansion of skill psychology into the regulation of social affairs, and that norms might be usefully explained by appealing the same kinds of anticipatory corrections first developed to produce lithic tools.

This is still a hypothesis. Nonetheless, Birch’s account provides an important platform for thinking about the emergence of cognitive novelties. Under this framework, cognitive novelties might be understood as routinized inferences or manipulations of informational vehicles, carried out in accordance with standards for correct manipulation. They may even continue to involve motor skills, as seems to be the case with AMC. And just as with motor skills, cognitive novelties can be understood as being the result of training regimes: long spans of effortful practice that take advantage of exogenous information and occasional tutelage in an environment engineered for epistemic transfer.

## Conclusion

The AMC case study involves not only the learning of algorithmic motor patterns and standards for correctly manipulating information-carrying vehicles, but the internalization of those vehicles in imagistic representations. It is this relationship—where the information form in the environment is *mirrored* in cognitive processing—that can generate cognitive novelties, and which structural-causal explanations target. My suspicion, though one I cannot pursue in great detail here, is that other cognitive novelties would also be illuminated by structural-causal explanations. Both Heyes and Sterelny (2003), for instance, have argued that mindreading capacities are learned, pointing to evidence that shows exposure to mental state lexical items is necessary for fully-fleshed mindreading capacities, and that competence correlates with exposure to narratives and explanations employing such mental state terms.^19^

The idea that cognition comes to mirror structure in the world is not new. Dennett (1991), for instance, makes a similar point when he discusses language acquisition. For Dennett, language installs a ‘virtual serial machine’ on the parallel processing circuity of the brain, tweaking “myriad microsettings in the plasticity of the brain.” (219) Like Heyes’s story, this is one where “relatively small hardware differences […] allow us to both create and benefit from public language and other cultural developments in ways that lead to a great snowball of cognitive change” (Clark 1997, p. 198). Nonetheless, even if the ideas here are not new, they take on added importance and salience within the niche construction approach. It is not just language that radically transforms human cognition, but potentially a whole host of culturally evolved gadgets. They do so by exploiting plastic, skill-oriented cognition to mirror the form of exogenous informational assemblages.

Let me conclude by noting where the analyses of this paper have not yet reached. I began by noting the broad framework of the niche construction approach, and promised to say something about the evolution of cognitive novelties. As I’ve argued, attending to informational form is a valuable investigatory strategy—at least when one is aiming to explore variety in cognitive capacities seen among contemporary human populations. Indeed, I have focused exclusively on extant populations. This leaves open whether the explanatory strategy outlined here can be projected back into the evolutionary record.

Both AMC and ASL demonstrate the demanding empirical and ethnographic work needed to reconstruct informational form. Nonetheless, the wealth of techniques for gaining access to past climates, materials, and techniques should make us optimistic that the current account can be extended deep into the human record. Experiments with contemporary populations to recreate the skills and skilled development of ancient techniques (e.g. Stout 2002, 2011; Stout et al. 2008); increasingly sophisticated tools of paleoclimatic, paleobotanical, and archaeological science, and; rich narrative accounts of paleoanthropological life together provide convergent lines of evidence that might allow the reconstruction of ancient informational forms.

There are benefits to doing so. As suggested above, structural-causal explanations illuminate the relationship between form and function, particularly the constraints of form on adaptive change. This means that the informational form appealed to in structural-causal explanations might also serve as a potent target for cultural evolution: irregular organization can be made regular; information can be better designed to match our sensory and motor capabilities, and; teaching and learning strategies can lead to more reliable acquisition and internalization. Artefacts in particular come into view as potent targets for investigation. As material loci for cumulative change—whether understood in terms of increased efficiency, manipulability, systematicity, or the like—artefacts are both stores of information and platforms for further tinkering, experimentation, and innovation. Over time, and in negotiation with plastic cognitive systems, artefacts can facilitate the emergence of cognitive novelties like literacy, numeracy, or AMC (Malafouris 2013; Overmann 2016). In short, taking informational form itself as an adaptive target opens up a new avenue for cultural evolutionary research—and further shows the value of structural-causal explanations for the study of human cognitive evolution.

## Footnotes

1 Key to such claims are the models and research from Boyd and Richerson (1985, 2005). Similar arguments can be found in Sterelny (2003) and Heyes (2018).

2 Classic articulations of coevolutionary relationships between social learning and information landscapes can be found in Henrich and Gil-White (2001) and Henrich and McElreath (2007), however the importance of such relationships for human evolution has been most comprehensively explored by Sterelny (2012).

3 For helpful summaries of the coevolutionary relationships between cognition and other traits, see: Tomasello (2014) and Henrich (2016)

4 This epistemic framing is especially prevalent in the work of cultural evolutionary researchers, as noted by Lewens (2015) and Buskell (*forthcoming*).

5 Links between demography, cultural change, and cultural sophistication have been modelled by Henrich (2004), Powell et al. (2009), Kolodny et al. (2015), and Fogarty and Creanza (2017).

6 Especially the works of Clark (1997, 2003, 2008); Hutchins (1995, 2010); Malafouris (2013); and Sutton (2010).

7 I leave open how ‘maturity’ should best be construed. A natural reading, in line with a link I draw to skills later in the paper, characterizes it in terms of proficiency or mastery. I thank an anonymous referee for pressing me on this issue.

8 Heyes’s (2019) few remarks on the subject have been skeptical of the explanatory significance of niche construction. Moreover, Heyes (2018) argues for a ‘force theory’; a generalized schematic for explaining human cognitive novelties. She takes this to be distinct from accounts which provide narratives of the phylogenetic or ontogenetic origins of novelties. She might thus resist being characterized as a niche construction researcher, not least because her force theory appeals to abstract features of cultural traits and processes (her ‘Campellian’ approach to cultural evolution, discussed below) rather than the activities of human agents.

9 For Heyes these include (at least) mind reading, selective social learning, language, and imitation.

10 I note that Heyes’s appeal to selectionist (‘Campbellian’) cultural evolution and cultural group selection is contentious (e.g. Lewens 2015; Morin 2019).

11 Is the underlying cognitive picture provided here committed to classical computational cognitivism? The distinction at issue, between cognitive mechanism and informational input, is what Haugeland (1998) calls a ‘family’ feature of representational approaches to cognition (as opposed to features of specific representational genera). As I see it, such generic representationalism is amenable to cognitivist, connectionist, and predictive processing treatments, and the points argued for here are meant to apply broadly. This is not to say that representationalism is uncontentious. Recent enactivist work, for instance, has developed interesting critiques of the explanatory power of representationalism (e.g. Malafouris 2013). I am grateful to two anonymous reviewers who pressed me on these issues.

12 It is worth noting that Love (2008) uses the terms ‘innovation’ and ‘novelty’ to refer to distinct functional and formal divergences respectively. I have not followed Love in this terminology to avoid confusion. Within the cultural evolutionary literature—which this paper draws upon—this distinction is not made, and ‘innovation’ is often operationalized to refer to any functional or formal change to a trait.

13 I note that the adaptationist line of thought articulated here is not committed to a unilineal view of human or cultural evolution as, say, articulated by classical sociocultural evolutionists (Stocking 1987) or the overtly racist theorists of the post-reconstruction United States of America (Baker 1998). Instead the adaptationist position at stake here is one where a wide variety of systematic causes (including cultural evolutionary ones) can generate cognitive mechanisms (which may arise through learning) that display means-ends fittedness for specific tasks, which themselves may vary from culture to culture. I thank an anonymous reviewer for voicing their concerns on this point.

14 For an overview of evolutionary developmental biology, see Carroll (2005). For an evocative concrete example of both the heterogeneity of elements appealed to and the complex causal relationships involved, see Martin et al. (2016) on the regulatory elements involved in the development of the crustacean limb.

15 For a sample list of questions and competition instructions, consult https://www.recordholders.org/en/events/worldcup/.

16 Heyes (2018) argues that children grown up in environments rich in structure and information; that they encounter a wealth of stimulus rather than a poverty of the stimulus. Suffice it to say, Heyes believes that data from developmental psychology refute *brute-causal* accounts where the origin of cognitive mechanisms or concepts are triggered by exogenous stimuli (Fodor 1981).

17 I note that this claim is contentious, in part because the researchers differ in how the explanatory target is characterized (a feature of the literature that Heyes (1993) has critiqued). Speaking generally, there is a tendency within the primatological research to study ‘imitative behavior’ which encompasses a broad range of observation-action relationships, including emulation, acquisition of ritualized behavior, the ‘automatic’ production of learned behavior, and a range of other social learning processes. For some recent reviews and evidence surrounding primate imitation, see Subiaul (2016) and Clay and Tennie (2018).

18 Imitation is taken to be regularly established in infants by six to eight months of age—though the precise timing is contentious (Oostenbroek et al. 2016).

19 Heyes distinguishes between *explicit* and *implicit* mindreading (Heyes and Frith 2014; Heyes 2018). Explicit mindreading capacities allow for reconstructions and deliberations over actions—both one’s own and other’s—using mental state terms. Implicit mindreading, by contrast, encompasses a suite of predispositions and mechanisms for orienting towards agents and intentional action. When Heyes and Sterelny argue that mindreading capacities are *learned* they mean explicit mindreading capacities.
